# An analysis of direct and indirect costs in Hidradenitis Suppurativa

**DOI:** 10.1002/ski2.306

**Published:** 2023-11-02

**Authors:** Claudine Howard‐James, Anne‐Marie Tobin

**Affiliations:** ^1^ Dermatology Department Tallaght University Hospital Dublin Ireland

## Abstract

**Background:**

Hidradenitis Suppurativa (HS) is a chronic inflammatory skin condition with recurrent nodules and abscesses that culminate in purulent discharge and scarring. It has significant physical, psychological and financial impact.

**Objectives:**

This study plans to analyse patient costs associated with HS. Direct costs include prescription items. Indirect or out‐of‐pocket costs include dressings, analgesia, and healthcare‐related travel costs. This study will also assess disease impact on quality‐of‐life (QOL).

**Methods:**

Patients with HS diagnosis attending dermatology OPD at our public tertiary centre were invited to participate. Ethical approval was secured, and informed consent was obtained. Participants completed an anonymous survey which was analysed to identify costs associated with HS as well as demographics and QOL impact.

**Results:**

A total of 25 patients completed the survey; median age was 29% and 80% were female. Median time from HS onset to diagnosis was 2 years, with 24% waiting >10 years to be diagnosed. In the past 3 months, 20% spent >€200 in both categories; prescription and non‐prescription items. In the non‐prescription category, 36% of patients reported expenditure >€100 in the past 3 months. Dressings were the most common out‐of‐pocket expense (in 15/25 patients), followed by analgesia and protective clothing. Attendance at medical appointments cost 24% of patients €50–€200. Four participants reported difficulty accessing HS treatments due to associated costs. Mean number of absence days from work/education as result of HS was 8.7 in the past 3 months. Two patients reported being on disability allowance, and two on unemployment benefit as result of their skin disorder. In the QOL question; 96% reported disease impact on QOL, and 11 participants reported that it affected their life ‘very much’.

**Conclusions:**

HS is a chronic inflammatory skin condition with significant financial burden alongside the well‐analysed biopsychosocial disease impact. Financial burden can be divided into direct prescription costs and indirect costs such as non‐prescription items, protective clothing and travel costs which we have explored in this study. Further research is needed in this area to identify and optimise both the financial and QOL implications of HS in acute flares and chronic disease management.

## Graphical abstract

Hidradenitis Suppurativa (HS) is a chronic inflammatory skin condition of pustular nodules, abscesses and scarring with significant biopsychosocial impact. This study explores the additional financial burden of HS in the domains of prescription and non‐prescription costs in the Irish public healthcare system by means of a survey, also analysing quality‐of‐life impact of the disorder. We found evidence of HS‐related expenditure both for prescription and non‐prescription items such as dressings, analgesia and protective clothing; adding to the quality‐of‐life impact and burden of dermatological disease.

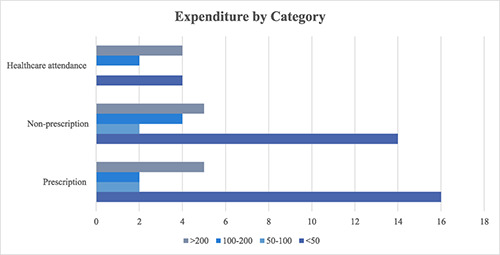

1

This qualitative research finds that Hidradenitis Suppurativa (HS) imposes significant financial burden in addition to biopsychosocial impact.

**What is already known about this topic?**

- HS is a chronic inflammatory skin condition with recurrent pustular lesions which lead to scarring, affecting approximately 1.4% of the Irish population. The impact on quality‐of‐life has been shown to be greater than other dermatological disorders due to the nature of the skin lesions, and it also imposes financial burden.

**What does this study add?**

- This study adds qualitative evidence of the financial implications of HS on patients in the Irish public healthcare system, subdivided into direct (prescription) and indirect costs such as dressings and analgesia, which are often unrecognised. As it is a disease characterised by acute flares and chronic skin changes, there is dual financial burden.

**How might this impact on clinical practice?**

- Clinical implications include the acknowledgement of financial burden, both direct and indirect, alongside the significant biopsychosocial impact of Hidradenitis Suppurativa, which helps clinicians provide a holistic approach to management.

## 2 INTRODUCTION

Hidradenitis Suppurativa (HS) is a chronic inflammatory skin condition characterised by recurrent boil‐like nodules and abscesses that culminate in a purulent discharge, sinuses, and scarring.^1^ It has significant physical, psychological and financial impact on those affected by the condition.

This study plans to analyse the direct and indirect patient costs associated with the management of HS. Direct costs include prescription items such as antibiotics and biologic agents. Indirect costs include over‐the‐counter products such as dressings and analgesia, and healthcare‐related travel costs. This study will also assess disease impact on quality‐of‐life (QOL).

## 3 MATERIALS AND METHODS

Patients with a diagnosis of HS attending dermatology outpatient clinic at our public tertiary centre were invited to participate in the study. Ethical approval was secured, and informed consent was obtained from participants. Participants completed an anonymous survey which was analysed to identify costs associated with HS as well as demographics and QOL impact.

## 4 RESULTS

**FIGURE 1:**
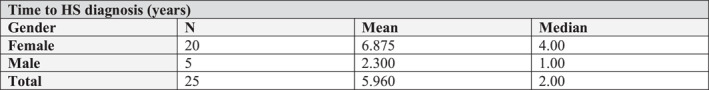
Table demonstrating mean and median time to diagnosis subdivided by gender.

**FIGURE 2:**
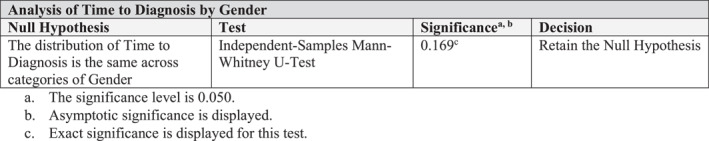
Analysis of time to diagnosis by gender, using Mann–Whitney *U*‐test.

A total of 25 patients completed the survey, of which the median age was 29 (range 19–61) and 80% (20) were female. Of those for whom Hurley Stage was known; 4% were Hurley I, 32% Hurley II and 40% Hurley III. The median time from HS onset to diagnosis was 2 years, with a mean of 5.96 years and one patient waiting 20 years for formal HS diagnosis, with six patients waiting >10 years to be diagnosed. Mean time to diagnosis was greater in females (6.875 years) than in males (2.3 years), although this was not statistically significant (*p* = 0.169), as shown in Figures 1 and 2.

Of this cohort, 22 had a medical card which covered some or all of the prescription costs. These include general medical cards; which entitle the holder to pay only a nominal cost of €1.50 per prescription item, and drug payment scheme cards; which entitle the holder to pay a maximum of €80 on prescription items per month.^2^

The survey analysed HS‐related expenditure in the past 3 months in the areas of prescription/direct costs, non‐prescription/indirect costs (which may also be referred to as out‐of‐pocket costs) and medical attendance costs.

**FIGURE 3:**
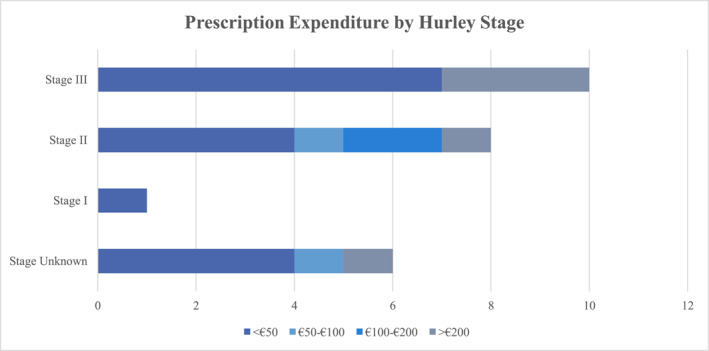
Bar chart of prescription expenditure subdivided to account for Hurley Stage, if known.

**FIGURE 4:**
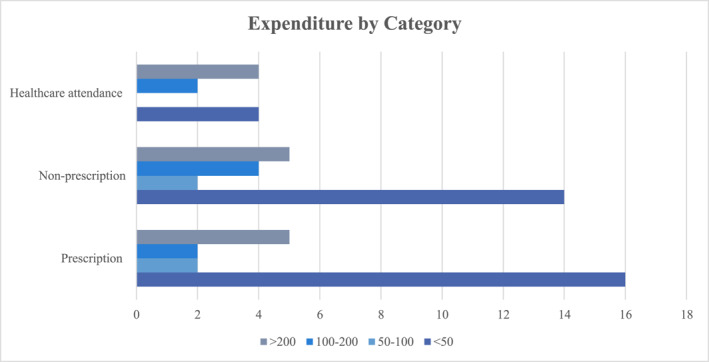
Bar chart demonstrating healthcare attendance costs, non‐prescription costs and prescription costs.

‐ A total of 16 patients spent < €50, 2 spent €50–100, 2 spent €100–200, and 5 spent >€200 on prescription expenditure related to their HS. Prescription costs were noted to be higher for those with Hurley Stage II‐III disease than those with Hurley Stage I HS, as shown in Figure 3, although Hurley Stage was not known for all patients.
‐ In the non‐prescription category: 14 patients spent <€50, 2 spent €50–100, 4 spent €100–200, 5 spent >€200. Breakdown of non‐prescription/indirect expenditure was subdivided into dressings (15 patients), antiseptic (4 patients), analgesia (9 patients) and other items such as protective clothing (9 patients).
‐ Attendance at medical appointments also entailed cost for seven patients, as outlined in Figure 4: 4 spent <€50, 2 spent €100–200, and one patient spent >€200 on travel to hospital or GP appointments.
‐ Four participants reported difficulty accessing HS treatments due to associated costs.

In an analysis of the impact of HS on career and education due to absence days; the median was 1 day, mean 8.7%, and 12% had >20 absence days (22, 30 and 90 days). Two patients reported being on disability allowance, and two on unemployment benefit as result of the disorder.

**FIGURE 5:**
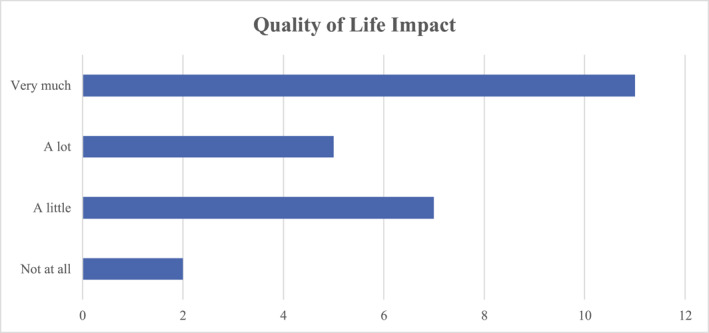
Bar chart analysis of the quality of life impact of Hidradenitis Suppurativa on surveyed patients.

Figure 5 demonstrates participants' answers when asked to assess how much their skin condition affected their life, 93% reported some disease impact on QOL, and 44% answered ‘very much’.

## 5 DISCUSSION

A recent systematic review and meta‐regression analysis found the overall prevalence of HS to vary greatly, but reports an overall population estimate of 0.40%,^3^ and quotes an estimated Irish figure of 1.4%.^4^ While the incidence may seem low, there is no doubt that disease impact on patients is high; with 23 out of 25 of participants in our study reporting QOL impact. It is a disorder with significant biopsychosocial implications, with a greater QOL impact than other dermatological disorders and a greater incidence of disease‐related morbidity such as depression.^5^

The evidence of the financial burden of HS is clear from this study. It is an aspect of the disorder that is not yet extensively evaluated, yet has huge impact on patients. Our study results are displayed in euro, which equates to 0.766 US dollars when purchasing power parities are applied.^6^ A 2014 analysis of healthcare utilisation costs focused mainly on emergency presentations in the setting of HS flares, but also found that mean outpatient costs were higher in HS than in psoriasis and control groups.^7^ As HS is a chronic disease with acute flares, it means that there is a dual burden of emergency presentations and long‐term disease management.

Of note, Ireland has a two‐tier healthcare system, divided into public Health Service Executive funded hospitals and privately funded hospitals which cater to patients with private health insurance.^8^ 47% of people in Ireland hold private health insurance, as of May 2023.^9^ Our study took place in a public hospital, meaning participants in the study were not utilising private health insurance to attend dermatology clinic. In addition, access to general medical cards which cover prescription costs is means tested in Ireland, meaning the holder's weekly income must be below a certain threshold to qualify.^2^ This study's data regarding medical cards which contribute towards prescription costs must also be interpreted in the context of public healthcare attendance, as 22 out of 25 participants in the study held medical cards. This data only provides an insight into financial burden of HS in patients utilising the public healthcare system and thus cannot be generalised to the entire population, given that almost half of the Irish population hold private health insurance.

This study demonstrates briefly the delay that can occur in HS diagnosis, with a median time from onset to diagnosis of 2 years and a mean of 5.96 years. This diagnostic delay has also been noted in larger systematic reviews.^3^ Interestingly, mean time to diagnosis was greater in females (6.875 years) than in males (2.3 years), although this was not statistically significant (*p* = 0.169). Factors which may contribute to the delay include its low prevalence and unfamiliarity of clinicians with the disorder as well as the social stigma and self‐esteem impact of pustular skin lesions.

Limitations of this study include the small sample size and subjectivity of participant answers regarding QOL impact and difficulty accessing treatments due to associated costs. As mentioned above, the study only analyses public patients and does not reflect financial burden of patients attending in a private healthcare setting. We did not categorise cost analysis into treatment of acute flares and chronic disease management, which both carry financial implications. We plan to reassess to include a larger sample size, further categorise costs into acute flares and long term management, and to utilise objective scoring tools such as Disease‐Related Quality of Life Index.

## 6 CONCLUSIONS

HS is a chronic inflammatory skin condition with significant financial burden alongside the well‐analysed biopsychosocial disease impact. Financial burden can be divided into direct prescription costs and indirect costs such as non‐prescription items, protective clothing and travel costs which we have explored in this novel qualitative study. Further research is needed in this area to identify and optimise both the financial and QOL implications of HS in acute flares and chronic disease management, both in public and private healthcare settings.

## CONFLICT OF INTEREST STATEMENT

The authors declare no conflicts of interest.

## AUTHOR CONTRIBUTIONS

**Claudine Howard‐James**: Conceptualization (equal); data curation (lead); formal analysis (lead); investigation (lead); methodology (lead); project administration (lead); writing—original draft (lead); writing—review and editing (lead). **Anne‐Marie Tobin**: Conceptualization (equal); supervision (lead).

## ETHICS STATEMENT

This study protocol was reviewed and approved by the SJH/TUH Joint Research Ethics Committee, approval number 2376. Written informed consent was obtained for participation in this study.

## Data Availability

All data generated or analysed during this study are included in this article. Further enquiries can be directed to the corresponding author.
